# CNAsim: improved simulation of single-cell copy number profiles and DNA-seq data from tumors

**DOI:** 10.1093/bioinformatics/btad434

**Published:** 2023-07-14

**Authors:** Samson Weiner, Mukul S Bansal

**Affiliations:** Department of Computer Science and Engineering, University of Connecticut, Storrs, CT 06269, United States; Department of Computer Science and Engineering, University of Connecticut, Storrs, CT 06269, United States; Institute for Systems Genomics, University of Connecticut, Storrs, CT 06269, United States

## Abstract

**Summary:**

CNAsim is a software package for improved simulation of single-cell copy number alteration (CNA) data from tumors. CNAsim can be used to efficiently generate single-cell copy number profiles for thousands of simulated tumor cells under a more realistic error model and a broader range of possible CNA mechanisms compared with existing simulators. The error model implemented in CNAsim accounts for the specific biases of single-cell sequencing that leads to read count fluctuation and poor resolution of CNA detection. For improved realism over existing simulators, CNAsim can (i) generate WGD, whole-chromosomal CNAs, and chromosome-arm CNAs, (ii) simulate subclonal population structure defined by the accumulation of chromosomal CNAs, and (iii) dilute the sampled cell population with both normal diploid cells and pseudo-diploid cells. The software can also generate DNA-seq data for sampled cells.

**Availability and implementation:**

CNAsim is written in Python and is freely available open-source from https://github.com/samsonweiner/CNAsim.

## 1 Introduction

Somatic copy number alterations (CNAs) are pervasive among human cancers and have been strongly linked with tumorigenesis, with upwards of 30% of the tumor genome being altered by CNAs alone (Harbers *et al.* 2021). In particular, pan-cancer studies show that 25% of the genome from a typical solid tumor is altered through whole-chromosomal and chromosome-arm CNAs, and an additional 10% is altered through many smaller focal CNAs (Beroukhim *et al.* 2010, Ben-David and Amon 2020), with some overlap. Furthermore, whole-genome duplication (WGD) has been reported to occur in no <30% of cancer genomes (Zack *et al.* 2013, Bielski *et al.* 2018).

In recent years, single-cell DNA-sequencing (scDNA-seq) technologies have enabled the study of somatic CNAs and mutations at the single-cell resolution (Navin 2014, Gao *et al.* 2016, 2017, Velazquez-Villarreal *et al.* 2020). Given the profound impact of CNAs in cancer, a wide array of tools have been developed for identifying and analyzing CNAs from scDNA-seq data (Mallory *et al.* 2020b) and for reconstructing tumor cell lineage trees based on single-cell copy number profiles (CNPs) (Cordonnier and Lafond 2020, Kaufmann *et al.* 2021, Wang *et al.* 2021, Hui and Nielsen 2022). As scDNA-seq data become increasingly available, there is a growing need for reliable simulation frameworks that can be used to assess the performance of such methods. This is particularly important given the high error rates in scDNA-seq data and a lack of ground truth datasets. In recent years, a number of simulators have been developed to address this challenge by modeling specific aspects of the scDNA-seq pipeline. These include PSiTE (Yang *et al.* 2019), SCSsim (Yu *et al.* 2020), SCSIM (Giguere *et al.* 2020), MosaicSim (Srivatsa *et al.* 2022), SCSilicon (Feng and Chen 2022), and SimSCSnTree (Mallory and Nakhleh 2022). Among these, SCSIM (Giguere *et al.* 2020) and SCSsim (Yu *et al.*, 2020) generate single-cell sequencing reads assuming biases from the whole-genome amplification (WGA) process, however neither approach models the evolutionary relationships between cells or subclones. MosaicSim (Srivatsa *et al.* 2022) is a more general next-generation sequencing (NGS) simulator tool that explicitly creates a phylogenetic tree of the tumor but is limited in the diversity of and user-control over possible CNAs. PSiTE (Yang *et al.* 2019) and SimSCSnTree (Mallory and Nakhleh 2022) are two phylogeny-aware simulators that implement a wide range of possible CNAs at the focal scale, but are limited at larger scales. The length of CNAs generated by PSiTE follows an exponential distribution, making chromosome-level CNAs extremely unlikely. While the same is true for SimSCSnTree, chromosomal CNAs are simulated separately; however they are always shared by the entire tumor population rather than individual subclones, and cannot occur on individual chromosome-arms.

A common thread among all aforementioned tools is that they are designed to generate synthetic sequencing reads where the set of variants and/or evolutionary relationships are known. However, this approach is not well-suited for the evaluation of single-cell analysis methods that rely on CNA/mutation data since it greatly limits the number of cells that can be simulated and, furthermore, requires users to first apply a variant calling or CNA detection tool. This limitation was partly addressed by CellCoal (Posada 2020), a tool that omits the generation of sequencing reads and outputs observed mutation profiles with error that reflects the biases of NGS and variant calling. However, CellCoal is only able to simulate SNVs and therefore cannot be used for generating CNPs. To the best of our knowledge, SCSilicon (Feng and Chen 2022) is the only existing simulator that is able to generate CNPs directly. However, there are significant drawbacks to the CNPs generated using SCSilicon. First, there are no underlying evolutionary relationships among cells or subclones, and copy number segments are generated outright. Second, the output CNPs do not adequately model errors resulting from single-cell sequencing or CNA detection algorithms (Mallory *et al.* 2020a).

In this work, we present CNAsim, an improved simulator for single-cell CNPs and DNA sequencing data. CNAsim addresses several limitations of existing simulators and offers significantly improved scalability, a high degree of customizability, and improved biological realism of simulated data. CNAsim has a number of unique features not available in any other simulator. Notably, CNAsim can:

1. Efficiently generate both ground truth CNPs and CNPs that mimic the expected error-prone output of CNA detection methods applied to single-cell data.
2. Generate CNAs at different scales including WGD, whole-chromosomal CNAs, and chromosome-arm CNAs.
3. Simulate subclonal population structure defined by the accumulation of chromosomal CNAs.
4. Dilute the sampled cell population with both normal diploid cells and pseudo-diploid cells.

In addition, CNAsim implements numerous enhancements that improve biological realism and utility, bringing together many features and best practices that were previously not simultaneously available in any single simulator. For example, CNAsim can (i) generate single-cell genealogies under the neutral coalescent with selective sweeps, (ii) apply a wide range of possible focal CNAs with tunable parameters, (iii) use user-provided reference genomes, and (iv) generate synthetic paired-end sequencing reads and CNPs. Table 1 identifies these and additional features and specifies which of those features are present or absent in existing simulators.

**Table 1.** Key features and functionality of CNAsim and other simulators capable of generating single-cell data.^a^

Tool	Year	Feature										
		Single-cell sequencing reads	Phylogenetic tree	Dynamic clonality	Normal cells	Pseudo-normal cells	Focal CNAs	Chromosomal CNAs	WGD	Outputs CNA/genotype profiles	CNA/genotype profile error model	Haplotype aware sequencing reads
SCSIM	2020	*✓*										
SCSsim	2020	*✓*					*✓*					
SCSilicon	2022	*✓*			*✓*		*✓*			*✓*	[*✓*]	
CellCoal	2020		*✓*							*✓*	*✓*	
PSiTE	2019	*✓*	*✓*	*✓*			*✓*					
MosaicSim	2022	*✓*	*✓*	*✓*			*✓*	[*✓*]				
SimSCSnTree	2022	*✓*	*✓*	*✓*			*✓*	[*✓*]				
CNAsim	2023	*✓*	*✓*	*✓*	*✓*	*✓*	*✓*	*✓*	*✓*	*✓*	*✓*	*✓*

a A check mark within brackets indicates that the feature is implemented partially or with limited functionality. An extended table with additional features appears in Supplementary Table S2.

## 2 Features and implementation

**Figure 1.**
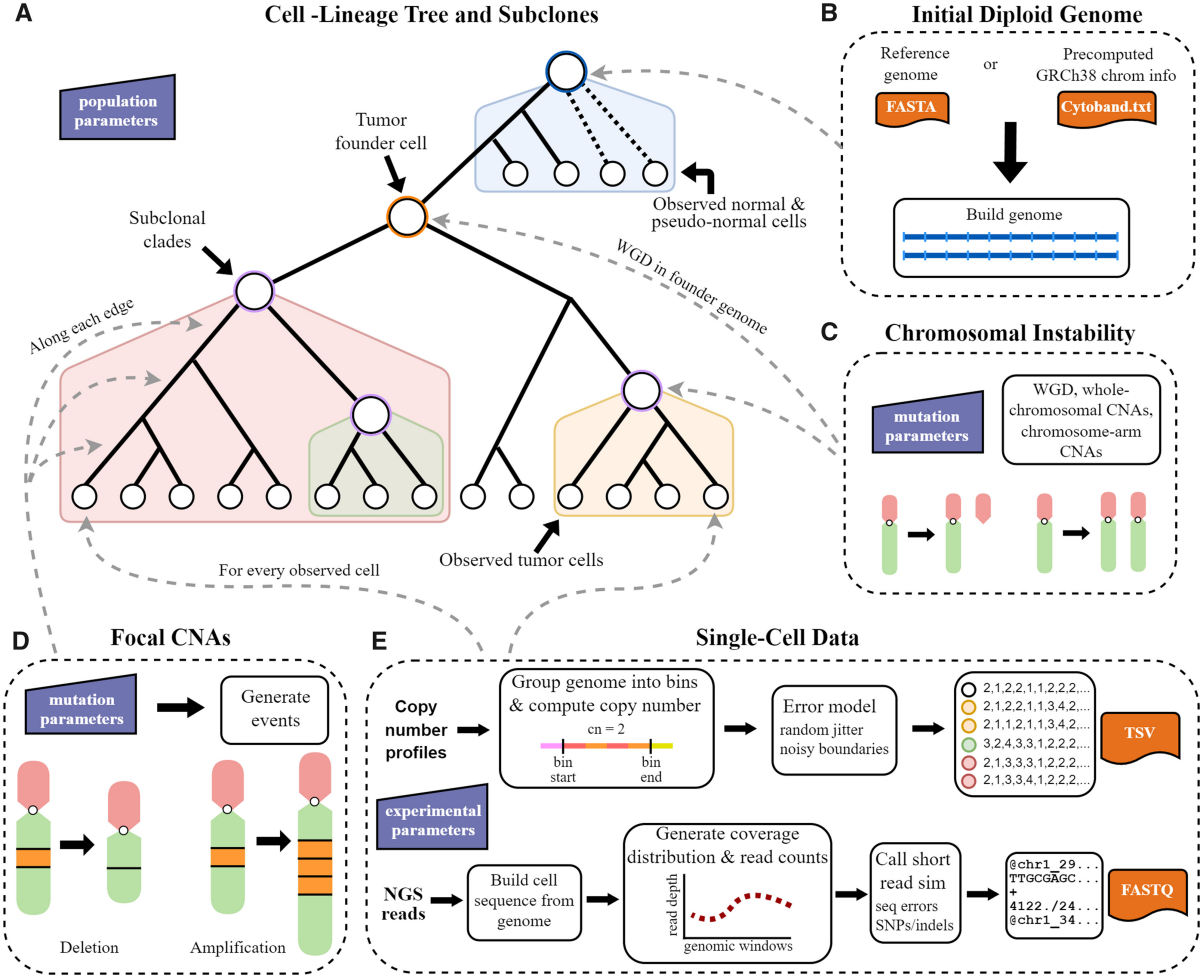
CNAsim features and key steps. (A) CNAsim first generates a cell lineage tree under neutral coalescence for all ancestral and observed cells in the experiment. Here, observed cells refer to all cells that are acquired at the time of sampling for downstream analysis. (B) Starting with a healthy diploid genome placed at the root of the tree, initialized from either an input reference genome or a precomputed human reference cytoband file, cell genomes are evolved along the branches by introducing somatic copy number aberrations. (C) Normal and pseudo-normal cells attached near the root of the tree progressively accumulate mutations until the tumor founder cell is created in a burst of genomic instability. Following this event, the primary tumor lineage is generated under neutral coalescence. Distinct clades within the tumor lineage are selected to represent diverging subclonal populations and undergo further chromosomal gains and losses. (D) All tumor cells and pseudo-normal are subject to smaller focal copy number aberrations, including gains, losses, and copy-neutral LOH, along the edges of the cell-lineage tree. (E) At the leaves of the tree, single-cell data is generated for the observed cells. This includes detailed CNPs that consider specific biases of single-cell sequencing (top workflow), as well as paired-end synthetic sequencing reads for each cell (bottom workflow).

CNAsim starts by simulating an exponentially growing tumor population under neutral coalescence (Hudson 2002), but with the option of selective sweeps leading to rapid bursts of coalescence. The observed cells in the experiment are sampled from a much larger tumor population at a specific point in time and whose evolutionary relationships are described by a cell lineage tree (Fig. 1A). See Supplementary Section S2 for additional details on the simulation of the cell lineage. The set of sampled observed cells can be diluted with fractions of normal and/or pseudo-normal cells existing outside of the tumor lineage, the latter of which is useful for simulating near-diploid cells that are unrelated to the major tumor populations, but have also diverged from the normal cells by a varying degree (Navin *et al.* 2011, Kim *et al.* 2018, Baslan *et al.* 2020, Konoshenko *et al.* 2020). See Supplementary Section S1 for implementation details of normal and pseudo-normal cells. Within the main tumor lineage, individual clades are selected to represent diverging subclonal populations whose genomic profiles harbor a significant degree of separation from the rest of the tumor population. The ancestral genome of each subclonal population achieves this separation by undergoing a unique set of whole-chromosomal and chromosome-arm CNAs. CNAs occurring at the chromosome level have been used previously to define tumor subclones (Velazquez-Villarreal *et al.* 2020), and is well suited for explaining recent scDNA-seq datasets (Secrier *et al.* 2016, Minussi *et al.* 2021, Zaccaria and Raphael 2021), but has yet to be implemented in any existing simulator. See Supplementary Section S3 for further details on subclonal populations.

CNAsim simulates the somatic evolution of a diploid genome by introducing CNAs along the edges of the cell lineage tree. For improved scalability, CNAsim utilizes a simplified genome representation that discretizes a reference sequence into an ordered set of uniform-sized regions. See Supplementary Section S4 for additional details on the simplified genome representation. Starting from an ancestral diploid genome at the root tree (Fig. 1B), which may either be simulated or provided by the user, individual focal CNA gains and deletions are processed along each edge in a top-down traversal (Fig. 1D). CNAsim models the length, location, type, and amplification magnitude with separate distributions each with tunable parameters (Itsara *et al.* 2010, Mallory *et al.* 2020a). Chromosome-level events, including both whole-chromosome and chromosome-arm CNAs, are added along the edges into and out of the ancestral founder cell of the entire tumor lineage, as well as the edges into subclonal ancestral cells (Fig. 1C). Additionally, the user can simulate a WGD at the onset of the tumor lineage. Unlike focal CNAs, all newly created chromosomes that occur as a result of WGD or whole chromosomal duplications are treated as new alleles, consistent with the underlying biological mechanisms (Knouse *et al.* 2017, Harbers *et al.* 2021). Supplementary Section S5 contains further implementation details for generating CNAs.

Once the genomes of all ancestral and sampled cells have been simulated, CNAsim generates single-cell data for the observed cells (Fig. 1E). One of the primary novelties of CNAsim is the direct simulation of CNPs that account for technical noise in the scDNA-seq pipeline. Copy number errors are introduced in two distinct ways. First, noise is introduced at the boundaries between distinct copy number segments, motivated from existing reports of noise concentrating at these areas in real CNPs (Garvin *et al.* 2015, Mallory *et al.* 2020a). Second, random fluctuations are added to the CNPs proportional to the individual copy number states, reflecting read count nonuniformity characteristic of scDNA-seq (Gawad *et al.* 2016). For additional details on generating CNPs and the error model, see Supplementary Section S6. CNAsim can also be used to generate single-cell sequencing reads that mimic the technical challenges and limitations of real single-cell sequencing technology. For each observed cell, CNAsim first converts the simplified mutated genome into a full DNA sequence by substituting each region with its corresponding reference segment (see Supplementary Section S4), which is then partitioned into nonoverlapping windows of fixed size. Each of the two alleles in the mutated genome can be assigned a separate haploid reference genome, enabling the simulation of haplotype-aware sequencing reads. CNAsim generates a smooth coverage distribution over the windows with a tunable degree of fluctuation (Mallory and Nakhleh 2022), which is used to draw read counts for each window. CNAsim makes use of a third party short-read simulator dwgsim (https://github.com/nh13/DWGSIM) to simulate NGS short reads. Further details on generating sequencing reads can be found in Supplementary Section S7.

**Performance and scalability.** Using a single processor, CNAsim can generate high-resolution whole-genome CNPs for thousands of cells in a few hours, and low-coverage whole-genome sequencing reads from hundreds of cells in well under a day. For the latter, CNAsim can easily be run in parallel leveraging multiple cores simultaneously for further reduction in running time. For both types of data, CNAsim requires minimal memory usage and storage space compared with existing simulators. Detailed results on running time appear in Supplementary Section S8.

## 3 Availability and requirements

CNAsim can be used on any platform with a Python interpreter through the command line. The core functionality of CNAsim requires only the installation of existing python packages and is sufficient to generate CNPs. Included among these Python packages are BioPython (Cock *et al.* 2009) and PyFaidx (Shirley *et al.* 2015) used for parsing sequence files, as well as msprime (Baumdicker *et al.* 2022) for an implementation of the population growth models. Generating sequencing reads with CNAsim requires the additional installation of samtools and DWGsim. CNAsim is open source under GNU GPLv3. The source code, detailed user manual, and sample commands are freely available from https://github.com/samsonweiner/CNAsim.

## 4 Discussion and conclusion

CNAsim is a new simulator for generating single-cell CNP and DNA-seq data. It includes a number of unique features and other enhancements that significantly improve scalability and biological realism. While CNAsim can facilitate the evaluation of a wide array of methods, it is particularly designed for those that use copy number data, study clonal compositions and large-scale CNAs, or infer copy number profiles and CNAs.

There are several features that can be added or expanded upon to further improve the versatility of CNAsim. First, expanding the types of mutations to include SNVs, indels, structural changes, and segmental insertions would provide a richer set of variations to the genome. Furthermore, the current approach of modeling chromosome-arm CNAs simplifies complex mechanisms such as breakage–fusion–bridge cycles and unbalanced translocations. It would be beneficial to expand the mutation model to include these mechanisms, along with rare mutations such as chromoplexy and kataegis. Second, while the error model implemented in CNAsim is effective at modeling general patterns of noise observed in real data, it could be improved to capture more specific error patterns such as those caused by read misalignment or poorly mappable regions. Third, additional types of data, such as bulk and exome-sequencing data, could be made available. Finally, single-cell data could be generated at various times during the cell-lineage to create temporal data.

## Supplementary Material

btad434_Supplementary_Data

## Data Availability

No new data were generated or analysed in support of this research. CNAsim software is available open-source from https://github.com/samsonweiner/CNAsim.
