# Supplementary material for: CNAsim: improved simulation of single-cell copy number profiles and DNA-seq data from tumors
Source: Bioinformatics. 2023 Jul 14;39(7):btad434. doi: 10.1093/bioinformatics/btad434 (PMC10363024; doi:10.1093/bioinformatics/btad434)
Supplement: btad434_Supplementary_Data [file btad434_supplementary_data.pdf]

# Supplementary Text

## S.1 Diluting sample with normal and pseudo-normal cells

A user-defined proportion of the sampled cells in the experiment can be taken from normal, healthy diploid cells outside of the tumor lineage. Samples taken from tumors of cancer patients are expected to contain a significant proportion of normal cells in addition to cancer cells [18]. Some experiments identify and discard normal cells before further downstream analysis through practices such as DAPI staining with flourescence-activated cell sorting [27, 43], however non-cancerous cells are often included [22]. In certain situations, normal cells play a crucial role in analysis, for example the SCOPE [62] method for CNA detection which uses normal cells as negative controls samples for read depth normalization. CNAsim dilutes the sampled population with normal cells by attaching observed cells directly to the root node with a special edge that contains no somatic mutations. Essentially, the added normal cells inherit an identical copy of the input genome placed at the root node.

In addition to normal cells, CNAsim can also simulate pseudo-normal cells. The presence of pseudo-diploid cells has been reported in existing scDNA-seq datasets [3, 30, 32, 44], and are typically defined as near-diploid cells that are seemingly unrelated to the major tumor populations, but have also diverged from the normal cells by a varying degree [44]. In CNAsim, pseudo-normal cells are introduced into the cell lineage tree along the edge between the root node and founder node of the primary tumor lineage. This edge represents the period of time in which the initial abnormal population of cells acquire the set of cancer causing mutations that result in neoplasm, and is therefore expected to have a significant number of focal CNAs compared to edges within the tumor lineage (see Supplemental Note S7). If $m$ is the total number of focal CNAs assigned to this edge, pseudo-normal cells are attached at random increments along the edge such that they inherit anywhere between 1 and $m - 1$ focal events.

The number of normal, pseudo-normal, and tumor cells is controlled by three parameters: the total number of sampled cells, the normal cell fraction, and the pseudo-normal cell fraction. The number of tumor cells is thus the remaining fraction, and is equivalent to the number of leaves in the main tumor lineage. By default, both fractions are set to 0.

## S.2 Simulation of tumor cell lineage tree

The main tumor lineage is simulated separately from the normal and pseudo-normal cells. CNAsim simulates an exponentially growing tumor population under neutral coalescence [28] where the observed tumor cells are sampled from a much larger population. The evolutionary relationships between the sampled tumor cells are represented as leaves in a cell lineage tree generated using the msprime package [4], and the common ancestor of all sampled tumor cells is referred to as the founder cell.

Neutral theory has been widely applied to, and observed in, tumor populations across cell types [5,9,35, 45,46,58,63] and is used in existing single-cell tumor phylogeny simulators [49,55,64]. However, the dynamics of neutral evolution have primarily been observed in cancer only after the accumulation of genomic changes that result in neoplasia, and it is believed that selection plays an important role in the initial stages of cancer progression. Therefore, it is assumed that the founder cell is sampled from a small existing population of cancer cells after oncogenic initiation.

While neutral evolution have been shown to dominate the genomic landscape during later stages of tumor growth [58,63], this does not necessarily imply the complete absence of selection [56]. In the context of tumor growth, selection among subclones may be a relevant factor in shaping the tumor landscape [54]. While a coalescent model for cancer subclone selection has yet to be fully developed [49], there have been attempts to model selection in the coalescent under limited scenarios, e.g., [4, 10]. Accordingly, CNAsim allows the user to introduce selection into the tumor lineage through a user-defined number of selective sweeps that result in a burst of rapid coalescence [4]. To achieve this, the population model alternates between a standard coalescence [28] and a structured coalescence with selective backgrounds [10] implemented in msprime [4] as the *SweepGenicSelection* ancestry model. However, since this approach likely does not adequately model subclonal selection, and since neutral evolution is an ideal null-model for comparison [12, 49], the default

number of sweeps in CNAsim is 0 (in other words, only the standard coalescence model is used). If the number of sweeps is $> 0$, each sweep is distributed randomly over the tumor lifespan, and the selective advantage of each sweep is controlled by a user-defined strength parameter [4].

CNAsim takes as input an exponential growth rate $\alpha$ used in the coalescence model. For convenience, we derive an appropriate value of $\alpha$ that is used as the default. We assume the tumor grows exponentially from a founder cell sampled from an initial population of $N_t = 1000$ that began $t$ generations in the past [11, 42, 61]. Diagnosis occurs in the present (at time $t = 0$) when the tumor reaches a population of $N_0 = 1 \times 10^9$ cells [5]. We approximate the time between onset and diagnosis to be 10 years [23, 41], during which we assume a cell turnover of once per day [5, 25]. The total number of generations $t$ is therefore $10 \times 365 = 3650$. This gives a growth rate of $\alpha = \left(\frac{\ln(N_t) - \ln(N_0)}{t}\right) \approx 3.785 \times 10^{-3}$.

## S.3 Clonal model

After the cell lineage tree is generated, a user-defined number of ancestral tumor cells can be selected to represent subclonal founders. In addition to focal CNAs, subclonal founder cells undergo a Poisson-distributed number of chromosomal events. All ancestral and observed cells that are descendants of a subclonal founder inherit its chromosomal aberrations, and are considered to be part of the same subclone. This approach of defining clones with chromosomal events is motivated by clonal structures observed in real scDNA-seq datasets [43, 51, 66], but was largely ignored in the era of bulk-sequencing [60].

Chromosomal events can also be added along the edges out of the founder cell to create a super-clonal structure over the distinct left and right branches. This feature is mimicking observations found in real data [66]. The number of chromosomal events for each super-clone is also Poisson-distributed, but has a separate mean from subclonal founders.

Subclonal founder cells are selected randomly among all ancestral nodes excluding the founder of the main tumor lineage. To ensure a uniform selection with respect to tree depth, the probability of choosing an ancestral node is proportional to the number of leaves in the subtree induced by the ancestral node. For stricter control over subclone size, ancestral nodes are chosen according to a user-defined Gaussian distribution. Specifically, the probability of selecting an ancestral node $n$ with $|n|$ descendant leaves is proportional to its density:

$$p(n) \propto \exp\left(-\frac{1}{2}\left(\frac{|n| - \mu}{\sigma}\right)^2\right) \tag{1}$$

where $\mu$ and $\sigma$ are the mean number of cells in a subclone and the standard deviation, respectively. The Gaussian distribution can be positively skewed by setting the value of $\mu$ to be sufficiently high with $\sigma$ acting as a skew parameter.

As an alternative to using the size of the induced subtree as a selection criteria, the probability of selecting an ancestral node can be made proportional to its branch length. Edge lengths in trees simulated under neutral coalescence often increase with tree height, ensuring a variety of both small and large subclones are selected.

## S.4 Simplified representation of genomes

Before simulating the evolution of cellular genomes along the cell lineage, CNAsim constructs an initial normal diploid genome which is placed at the root of the tree. This genome is equivalent to one copy each of the provided input haploid reference genomes, for two copies total. We note that passing two reference genomes is only necessary is one is interested in generating sequencing reads with known variants on a particular haplotype (see supplementary section S7). If a single haploid reference is provided, then the genome is equivalent to two identical copies of the provided reference. Rather than treating the initial genome as a long sequence of nucleotides, CNAsim partitions the sequence into an ordered array of non-overlapping regions of uniform length equal to $M$. Each region is represented by a unique integer identifier, corresponding to a particular $M$-length DNA segment of the reference sequence. For example, a reference

genome consisting of a single chromosome containing $1 \times 10^8$ bp and $M = 1000$ would be represented by two identical ordered vectors $\langle 1, 2, ..., 100000 \rangle$. When a CNA is generated along an edge of the tree and alters the inherited genome, this has the effect of augmenting the ordered array of regions. Conceptually, this is equivalent to an alteration of the corresponding DNA segment defined by each effected region. For example, if $M = 1000$, a 3000 bp deletion event starting at region 8 in the genome $\langle 1, ..., 6, 7, 8, 9, 10, 11, 12, ..., 100000 \rangle$ would result in $\langle 1, ..., 6, 7, 11, 12, ..., 100000 \rangle$. Using this condensed representation, the ordering and length of a genome is dynamically updated, which implicitly allows for overlapping events. Additionally, this representation naturally lends itself to generating ground truth copy number profiles through examining the number of regions assigned to each bin.

When choosing a value for $M$, one has to consider the trade-off between computational efficiency and biological realism. In any case, an appropriate lower bound for $M$ is the smallest scale at which a CNA can alter a genome. It is unclear where to draw the boundary between indels and CNAs, reflected in many different definitions of the lower bound for CNA size in literature. Typically, a variant has to be larger than 1000 base pairs to be classified as a CNA [15, 48, 53, 59], but minimum sizes have been reported at as small as 50 base pairs [37, 47]. Naturally, larger values of $M$ will improve the runtime of CNAsim and may be suitable for experiments with a large number of observed cells.

When partitioning each chromosome of the initial genome into regions, the number of regions is based solely on chromosome length. Chromosome lengths can be determined by the primary haploid reference genome passed by input from the user. This is a required input if CNAsim is used to generate sequencing reads, because a complete DNA sequence for each chromosome is needed for sampling reads. After the genomes are mutated along the cell-lineage tree, when generating sequencing reads for a particular cell, the genome is first converted back into a full sequence using the reference. More specifically, given the simplified genome representation, a complete sequence is built by substituting each region into its corresponding $M$-length nucleotide sequence from the provided reference genome(s). If on the other hand CNAsim is used only to generate CNPs, an input reference genome is not necessary. Instead, the user has the option to select chromosome lengths derived from the human reference genome GrCH38. Additionally, there are parameters to directly control the number of chromosomes and chromosome length. If CNAsim is used to simulate chromosome-arm events, the location of the centromere must be specified. Because the ratio of short to long arm of each chromosome cannot be learned from an input reference genome, users have two options when run in either mode. First, users can use chromosome arm ratios derived from GrCH38. Otherwise, a fixed ratio can be set for all chromosomes.

## S.5 CNA rates and properties

CNAsim can generate four types of CNAs, which are distinguished by size: focal CNAs, chromosome-arm CNAs, whole-chromosomal CNAs, and whole-genome duplication (WGD). The latter three are characterized as aneuploidy events and may only occur in select locations in the tree. On the other hand, focal CNAs can occur along all edges except those between normal cells and root. The number of focal CNAs along an edge is drawn from a Poisson distribution with a user-defined mean of $\lambda$. The value of $\lambda$ can either be fixed for each edge, or it can be proportional to edge length. In the latter case, $\lambda$ corresponds to the mean number of events for the average leaf edge length. Given that trees simulated under neutral coalescence generally have shorter edge lengths towards the leaves, it is expected that more events will be placed higher in the tree. Single-cell analysis of multiple triple-negative breast cancer samples and a melanoma tumor-line revealed the presence of many tens to even hundreds of CNAs in the genome of an individual cell [19, 43, 60]. These findings are consistent with pan-cancer studies across thousands of samples and multiple cancer types [7, 17, 26, 67], albeit with varying sequencing technologies and CNA detection methods. Furthermore, these studies may be under-reporting the true number of CNAs due to resolution constraints or simply filtering out smaller variants. To both simulate an appropriate number of events and ensure adequate genomic variation among observed cells, the value for $\lambda$ should reflect the total number of cells in the experiment. For an experiment with $n$ observed tumor cells, the expected number of CNAs accumulated in total for each observed cell is roughly $\log n * \lambda$.

Focal CNAs are chosen to be deletions or amplifications with a binomial distribution. Previous studies

have reported either a relatively equal number of deletions and amplifications, or a slightly greater proportion of amplifications [7, 67]. In either case, the number of deletions tends to scale with the number of amplifications [26], so by default the parameter in the binomial $p = 0.5$. Amplification events draw an additional number of copies to be gained from a geometric distribution [38]. The default parameter in the geometric is set to $p = 0.5$ for a mean of $1/p = 2$ additional copies based on the observation that the amplitude of most copy number gains is relatively low and higher copy number gains are rare [34, 36]. All additional copies are placed in tandem with the original. The length of a focal CNA is drawn from an exponential distribution with a mean of $\beta$ and a user-defined minimum length. Several probes into somatic CNA patterns have indicated that the frequency at which focal CNAs occur is inversely proportional to their sizes [7, 29, 33, 67]. However, mean CNA lengths appear to vary significantly across experiments; while some studies have reported average lengths less than 1 Mbp [1, 2], others have found this value to be multiple Mbp [7, 16, 20, 26, 67]. This variance can be partially explained by inconsistent terminology and a difference in technology and resolution. Setting $\beta = 5Mbp$ by default is well within existing estimates and is sufficient for meaningful evaluation. We note that in using the simplified genome representation (see Supplementary Section S4), focal CNA lengths must be scaled into units of $M$-length regions. That is, an event of length $X$ bp will modify $X/M$ regions, rounded to the nearest integer with a minimum of 1 region.

In choosing the location where a focal CNA occurs, first the allele-specific chromosome is chosen with probability proportional to the chromosome length. Then, a starting coordinate is chosen uniformly among all available positions. That is, for a chromosome of length $L$, the starting coordinate of an event of length $X$ is chosen uniformly among $\langle 1, ..., L - X \rangle$. We note that once the chromosome is selected, we ensure the event length is no greater than the chromosome length. To illustrate how focal CNAs alter the simplified genome, consider a default chromosome of length 100 Mbp with a region size of 1000 bp, represented by the vector $\langle 1, 2, ..., 100000 \rangle$. A 5000 bp deletion starting at the 10000 bp coordinate would alter the chromosome to be $\langle 1, ..., 9, 15, ..., 100000 \rangle$. If instead there was a 5000 bp duplication starting at the 10000 bp coordinate, the altered chromosome would be $\langle 1, ..., 9, 10, 11, 12, 13, 14, 10, 11, 12, 13, 14, 15, ..., 100000 \rangle$.

Recent studies into ITH have indicated that driver mutations leading to clonal sweeps rarely occur at intermediate or later stages in tumor proliferation without external factors [13], suggesting a considerable number of mutations initiate cancer growth and are pervasive across subclones [19, 50, 54]. To account for this, the value of $\lambda$ is amplified along the edge between the root node and founder node by a user-defined scalar. A whole-genome duplication (WGD) event can be placed along this edge after focal CNAs have mutated the founder genome. This is based on evidence for WGDs arising early in carcinogenesis across many cancer types, after the accumulation of transforming driver mutations [8].

There are three locations in the tree where chromosomal CNAs can occur: the edge between root and founder, the edges out of the founder, and edges above clonal nodes. The number of events selected along these edges are Poisson distributed with a separate mean for each location, denoted by $\alpha_1$, $\alpha_2$, and $\alpha_3$, respectively. Tuning these parameters, along with the option of adding WGD, gives the user significant control over the mutational landscape of the population and average ploidy. For example, a population-wide ploidy of three can be achieved by toggling WGD and using a high rate of chromosomal deletions above the founder. Data from pan-cancer studies show that 25% of the genome from a typical solid tumor is altered through whole-chromosomal and chromosome arm CNA [6]. To this effect, the default values for $\alpha_1$, $\alpha_2$, and $\alpha_3$ are such that the genome of the average observed cell in the population has undergone a median of 8 events.

Chromosomal CNAs are chosen to affect either a chromosome arm or a whole chromosome by a Binomial distribution. These two sources of large scale alteration are thought to have different underlying biological mechanisms [26, 31], and thus likely occur at different rates. Indeed, chromosome-arm CNAs are thought to occur more commonly than whole chromosomal CNAs [7, 57], and may also be the result of several focal CNAs. By default, chromosome CNAs are chosen to be arm-level events with probability $p = 0.75$. It should be noted that short and long arms are selected with equal probability. Both chromosome-arm CNAs and whole-chromosomal CNAs can either be a deletion or duplication event, but not an amplification [7]. The probability that the event is a deletion or duplication is according to a Binomial distribution. The probability of selecting a deletion, by default, depends on whether or not WGD is included. The pan-cancer

study of [67] found that the average ploidy in tumors with WGD was 3.31 instead of 4, whereas tumors without WGD had an average ploidy of 1.99. This suggests large amounts of loss are associated with WGD, whereas diploid genomes maintain a relative balance of gain and loss. In the presence of WGD, chromosomal CNAs are duplications with probability $p = 0.2$, otherwise $p = 0.5$

Each chromosome that was created as a result of WGD or whole chromosomal duplications are treated as new alleles. Unlike focal CNAs, which occur in tandem with the original segment, WGD and whole-chromosomal duplications are the result of errors during segregation [26, 31]. This implementation approach ensures consistency of any subsequent CNAs across the affected region.

## S.6 Generating copy number profiles

When run under CNP mode, CNAsim generates copy number data directly without the need of applying CNA detection algorithms to sequencing data. First, the $M$ length regions of the starting diploid genome are grouped into larger fixed size bins. The choice of bin size depends on the expected coverage of the experiment and the resolution of the copy number detection method, for example on the order of 500 kbp [21] to 5 Mbp [66]. The default bin size is set to 1 Mbp. Each observed cellular genome is 'mapped' back to the starting diploid genome by counting the number of copies of original $M$ length regions. Then, the copy number of each bin is computed to be the average number of occurrences of all regions assigned to that bin. This results in the desired cell by bin copy number profile matrix.

Despite recent advancements in CNA detection algorithms, obtaining accurate copy number profiles remains unreliable [38]. CNAsim implements a realistic noise model designed to capture error expected of real copy number profiles. The simulator models two independent sources of error at different steps in the single-cell DNA sequencing pipeline that may result in artifacts in the final copy number profiles. In scDNA-seq data, existing methods have reported a significant concentration of errors in the copy numbers occurring at the boundaries between distinct copy number segments where CNAs have occurred [21, 38]. This is likely due to the fact that low and/or non-uniform coverage can blur the precise endpoints between segments. CNAsim captures this noise pattern as part of the *boundary* model. The idea is to extend the boundaries of contiguous segments with the same copy number such that a random number of neighboring bins are assigned the segment's copy number. Conversely, the boundaries may be shrunk such that bins at the edges of the segment are assigned the copy number of the neighboring segment. For each chromosome of each cell, we iterate across the copy number profile while keeping track of the length of the current segment. When a shift occurs, we draw the new length of the segment from a Gaussian distribution with mean equal to the segment length, and a standard deviation equal to the segment length multiplied by an error rate $r_b$, given by the user.

Next, random jitter is introduced to each bin independently as part of the *jitter* model. The intention is to mimic random fluctuations in read counts across the genome that are common to scDNA-seq technologies, the primary cause being non-uniform coverage. The challenge of working with noisy sequencing data has been addressed in recent years, as CNA detection algorithms specifically designed for scDNA-seq data usually apply a normalization routine [39, 62]. However, many commonly used methods are still susceptible to fluctuations in read counts, while others are refactored methods originally designed for other sources of data like bulk sequencing [52]. For each bin with copy number $c$, a new copy number is assigned based on a draw from a Gaussian distribution with mean to equal $c$ and standard deviation equal to $c * r_j$, where $r_j$ is the error rate given by the user.

Default values of $r_b$ and $r_j$ were chosen by observing the error rates of the various CNA detection methods from the benchmarking study of [38], which reported precision and recall values for three different methods over various experimental conditions. While the results of these experiments appeared to vary greatly across different sequencing technologies and ploidies, it was reported that the best performing method achieved precision in the range $0.4 - 0.75$, and recall in the range $0.5 - 0.8$. To choose appropriate defaults, we evaluated precision and recall values of all combinations of $r_b \in \{0.01, 0.02, 0.04, 0.06, 0.08, 0.1, 0.12\}$ and $r_j \in \{0.05, 0.075, 0.1, 0.125, 0.15\}$. We found that setting $r_b = 0.04$ and $r_j = 0.1$ results in a precision and recall of 0.738 and 0.784, respectively, which correspond to the upper end of the reported ranges of [38].

## S.7 Generating sequencing data

CNAsim can be used to generate sequencing reads of each observed cell. To generate reads for an observed cell, the mutated genome is first expanded from the simplified representation into a complete sequence by replacing regions with their corresponding DNA segment in the reference. If a single haploid reference is provided, then the same region on either allele will be substituted for the same corresponding nucleotide sequence. If two haploid reference genomes are provided, which we refer to as the paternal and maternal reference, then the region on the paternal (resp. maternal) allele will be substituted for the corresponding nucleotide sequence from the paternal (resp. maternal) reference genome. This feature is meant to enable the evaluation of computational methods involving phasing, for example CHISEL [66], a method for inferring allele- and haplotype-specific copy numbers. CNAsim next determines the number of reads assigned to the cell. If uniform coverage is used, the genome-wide expected readcount $r$ is computed as $r = \frac{|G|*C}{2*L}$, where $|G|$ is the total length of the genome, $C$ is the coverage, and $L$ is the read length. The actual read count is sampled from a Poisson distribution with a mean of $r$. Paired-end sequencing reads are then generated using the short-read simulator dwgsim (https://github.com/nh13/DWGSIM) which requires a readcount and the mutated reference to execute.

If non-uniform coverage is used, the genome is partitioned into non-overlapping windows of fixed size, and readcounts are generated for each window. This is necessary because dwgsim samples reads uniformly across the provided reference. For a window of size $W$, the expected number of reads $r' = \frac{W*C}{2L}$. To simulate the effects of non-uniform read depth, an initial set of windows at some set interval $I$ sample a coverage coefficient $c_w$. The value of $c_w$ for the initial set of windows is drawn from a Beta distribution, scaled to be a value in the range $(0, 2)$, where a value of $c_w = 1$ indicating that window $w$ has exactly the expected read count. As for the remaining windows, a cubic bezier curve is generated over the entire set of windows seeded with the values drawn from the Beta distribution. This is to ensure that read counts change smoothly across windows and avoids jagged peaks.

The resulting read count of window $w$ is drawn from a Poisson distribution with mean $r' * c_w$. The parameters used in the Beta distribution control the degree of non-uniformity across windows. For modeling this, we follow the approach of SimSCSnTree [40], where a point on the lorenz curve is used to represent the coverage non-uniformity of the desired sequencing technology. The lorenz curve represented by that point is then transformed into the Beta distribution. For example, a value of $x = 0.5$ and $y = 0.27$ will model the coverage non-uniformity of MALBAC [38].

## S.8 Performance

We evaluated the resource requirements for generating sequencing data with CNAsim over three experimental conditions. Specifically, for each experiment we measured the total runtime, peak memory usage, and the final storage size. The results are shown in Table S1. Each experiment used the GRCh38 human reference genome assembly and a read length of 150, with all other parameters excluding those listed in the table remained at their defaults. For some of the experiments, we also evaluated the resource requirements of SimSCSnTree [40] and MosaicSim [55]. All experiments were run on an Intel Xeon 2.1 GHz processor with 64 GB of RAM using a single core, unless stated otherwise.

| | | CNAsim (uniform cov) | CNAsim (non-uniform cov) | SimSCSnTree | MosaicSim |
|---|---|---|---|---|---|
| 10 cells 0.1x coverage | runtime | 82 min | 115 min | 35 hr | 62 min |
| | memory | 4.25 Gb | 440 Mb | 8.8 Gb | 25.51 Gb |
| | storage | 2.5 Gb | 2.4 Gb | 13 Gb | 162 Gb |
| 100 cells 0.1x coverage | runtime | 14.7 hr | 20.2 hr | 376 hr | |
| | memory | 6.01 Gb | 528.8 Mb | 14.21 Gb | |
| | storage | 25 Gb | 25 Gb | 133 Gb | |
| 3 cells 30x coverage | runtime | 39.7 hr | 39.5 hr | | |
| | memory | 4.24 Gb | 436 Mb | | |
| | storage | 217 Gb | 212 Gb | | |

Table S1: Simulator Performance

We repeated the 100 cell, 0.1x coverage experiment using CNAsim with non-uniform coverage, this time making use of 8 parallel cores. This resulted in a reduction in runtime from 20.2 hr with a single core to 4.1 hr with 8 cores.

Next, we evaluated the resource requirements of using CNAsim to generate copy number profiles only. We found that a whole-genome 10000 cell dataset with a region length of 1000 bp took approximately 6 hours on a single core and used a negligible amount of memory and storage. It should be noted that the runtime scales linearly with both the number of cells and the region length. For example, a whole-genome 1000 cell dataset with a region length of 1000 bp will have an equivalent runtime to a whole-genome 10000 cell dataset with a region length of 10000, each taking approximately 35 minutes to execute on a single core.

## Supplementary Tables

| Feature | Tool | | | | | | | |
|---|---|---|---|---|---|---|---|---|
| | SCSIM | SCSsim | SCSilicon | CellCoal | PSiTE | MosaicSim | SimSCSnTree | CNAsim |
| Models WGA | ✓ | ✓ | ✓ | ✓ | | ✓ | | |
| NGS-specific error | ✓ | ✓ | ✓ | ✓ | ✓ | ✓ | ✓ | ✓ |
| Non-uniform coverage | | | | | | | ✓ | ✓ |
| Single-cell sequencing reads | ✓ | ✓ | ✓ | | ✓ | ✓ | ✓ | ✓ |
| Multiomic sequencing data | ✓ | | | | ✓ | ✓ | ✓ | |
| Phylogenetic tree | | | | ✓ | ✓ | ✓ | ✓ | ✓ |
| Dynamic clonality | | | | | ✓ | ✓ | ✓ | ✓ |
| Normal cells | | | ✓ | | | | | ✓ |
| Pseudo-normal cells | | | | | | | | ✓ |
| SNVs | ✓ | ✓ | ✓ | ✓ | ✓ | ✓ | ✓ | |
| CNAs | | ✓ | ✓ | | ✓ | ✓ | ✓ | ✓ |
| Diverse and tunable CNA properties | | [✓] | | | ✓ | | ✓ | ✓ |
| Whole chromosomal CNAs | | | | | | [✓] | [✓] | ✓ |
| Chromosome-arm CNAs | | | | | | | | ✓ |
| WGD | | | | | | | | ✓ |
| Generates variants in-situ | ✓ | | ✓ | ✓ | ✓ | ✓ | ✓ | ✓ |
| Outputs mutation profiles | | | ✓ | ✓ | | | | ✓ |
| Mutation profile error model | | | [✓] | ✓ | | | | ✓ |
| Haplotype-aware sequencing reads | | | | | | | | ✓ |

Table S2: An expanded list of features implemented in CNAsim and other relevant simulators for single-cell data. From left to right, these are SCSIM [24], SCSsim [65], SCSilicon [14], CellCoal [49], PSiTE [64], MosaicSim [55], and SimSCSnTree [40]. A check mark within brackets indicates that the feature is implemented partially or with limited functionality.

# References


[1] S. Agarwala, A. M. Veerappa, and N. B. Ramachandra. Identification of primary copy number variations reveal enrichment of calcium, and mapk pathways sensitizing secondary sites for autism. *Egyptian Journal of Medical Human Genetics*, 21(1):55, Dec 2020.

[2] J. S. Bae, H. S. Cheong, L. H. Kim, S. NamGung, T. J. Park, J.-Y. Chun, J. Y. Kim, C. F. A. Pasaje, J. S. Lee, and H. D. Shin. Identification of copy number variations and common deletion polymorphisms in cattle. *BMC Genomics*, 11(1):232, Apr 2010.

[3] T. Baslan, J. Kendall, K. Volyanskyy, K. McNamara, H. Cox, S. D'Italia, F. Ambrosio, M. Riggs, L. Rodgers, A. Leotta, J. Song, Y. Mao, J. Wu, R. Shah, R. Gularte-Mérida, K. Chadalavada, G. Nanjangud, V. Varadan, A. Gordon, C. Curtis, A. Krasnitz, N. Dimitrova, L. Harris, M. Wigler, and J. Hicks. Novel insights into breast cancer copy number genetic heterogeneity revealed by single-cell genome sequencing. *eLife*, 9:e51480, may 2020.

[4] F. Baumdicker, G. Bisschop, D. Goldstein, G. Gower, A. P. Ragsdale, G. Tsambos, S. Zhu, B. Eldon, E. C. Ellerman, J. G. Galloway, A. L. Gladstein, G. Gorjanc, B. Guo, B. Jeffery, W. W. Kretzschumar, K. Lohse, M. Matschiner, D. Nelson, N. S. Pope, C. D. Quinto-Cortés, M. F. Rodrigues, K. Saunack, T. Sellinger, K. Thornton, H. van Kemenade, A. W. Wohns, Y. Wong, S. Gravel, A. D. Kern, J. Koskela, P. L. Ralph, and J. Kelleher. Efficient ancestry and mutation simulation with msprime 1.0. *Genetics*, 220(3):iyab229, 2022.

[5] N. Beerenwinkel, T. Antal, D. Dingli, A. Traulsen, K. W. Kinzler, V. E. Velculescu, B. Vogelstein, and M. A. Nowak. Genetic progression and the waiting time to cancer. *PLoS computational biology*, 3(11):e225–e225, Nov 2007.

[6] U. Ben-David and A. Amon. Context is everything: aneuploidy in cancer. *Nature Reviews Genetics*, 21(1):44–62, Jan 2020.

[7] R. Beroukhim, C. H. Mermel, D. Porter, G. Wei, S. Raychaudhuri, J. Donovan, J. Barretina, J. S. Boehm, J. Dobson, M. Urashima, K. T. Mc Henry, R. M. Pinchback, A. H. Ligon, Y.-J. Cho, L. Haery, H. Greulich, M. Reich, W. Winckler, M. S. Lawrence, B. A. Weir, K. E. Tanaka, D. Y. Chiang, A. J. Bass, A. Loo, C. Hoffman, J. Prensner, T. Liefeld, Q. Gao, D. Yecies, S. Signoretti, E. Maher, F. J. Kaye, H. Sasaki, J. E. Tepper, J. A. Fletcher, J. Tabernero, J. Baselga, M.-S. Tsao, F. Demichelis, M. A. Rubin, P. A. Janne, M. J. Daly, C. Nucera, R. L. Levine, B. L. Ebert, S. Gabriel, A. K. Rustgi, C. R. Antonescu, M. Ladanyi, A. Letai, L. A. Garraway, M. Loda, D. G. Beer, L. D. True, A. Okamoto, S. L. Pomeroy, S. Singer, T. R. Golub, E. S. Lander, G. Getz, W. R. Sellers, and M. Meyerson. The landscape of somatic copy-number alteration across human cancers. *Nature*, 463(7283):899–905, Feb 2010.

[8] C. M. Bielski, A. Zehir, A. V. Penson, M. T. A. Donoghue, W. Chatila, J. Armenia, M. T. Chang, A. M. Schram, P. Jonsson, C. Bandlamudi, P. Razavi, G. Iyer, M. E. Robson, Z. K. Stadler, N. Schultz, J. Baselga, D. B. Solit, D. M. Hyman, M. F. Berger, and B. S. Taylor. Genome doubling shapes the evolution and prognosis of advanced cancers. *Nature Genetics*, 50(8):1189–1195, Aug 2018.

[9] I. Bozic, J. M. Gerold, and M. A. Nowak. Quantifying clonal and subclonal passenger mutations in cancer evolution. *PLOS Computational Biology*, 12(2):1–19, 02 2016.

[10] J. M. Braverman, R. R. Hudson, N. L. Kaplan, C. H. Langley, and W. Stephan. The hitchhiking effect on the site frequency spectrum of DNA polymorphisms. *Genetics*, 140(2):783–796, June 1995.

[11] T. Buder, A. Deutsch, B. Klink, and A. Voss-Böhme. Patterns of tumor progression predict small and tissue-specific tumor-originating niches. *Frontiers in Oncology*, 8, 2019.

[12] V. L. Cannataro and J. P. Townsend. Neutral Theory and the Somatic Evolution of Cancer. *Molecular Biology and Evolution*, 35(6):1308–1315, 04 2018.

[13] W. Cross, M. Kovac, V. Mustonen, D. Temko, H. Davis, A.-M. Baker, S. Biswas, R. Arnold, L. Chegwidden, C. Gatenbee, A. R. Anderson, V. H. Koelzer, P. Martinez, X. Jiang, E. Domingo, D. J. Woodcock, Y. Feng, M. Kovacova, T. Maughan, R. Adams, S. Bach, A. Beggs, L. Brown, F. Buffa, J.-B. Cazier, A. Blake, C.-H. Wu, E. Chatzpili, S. Richman, P. Dunne, P. Harkin, G. Higgins, J. Hill, C. Holmes, D. Horgan, R. Kaplan, R. Kennedy, M. Lawler, S. Leedham, U. McDermott, G. McKenna, G. Middleton, D. Morton, G. Murray, P. Quirke, M. Salto-Tellez, L. Samuel, A. Schuh, D. Sebag-Montefiore, M. Seymour, R. Sharma, R. Sullivan, I. Tomlinson, N. West, R. Wilson, M. Jansen, M. Rodriguez-Justo, S. Ashraf, R. Guy, C. Cunningham, J. E. East, D. C. Wedge, L. M. Wang, C. Palles, K. Heinimann, A. Sottoriva, S. J. Leedham, T. A. Graham, I. P. M. Tomlinson, and T. S. Consortium. The evolutionary landscape of colorectal tumorigenesis. *Nature Ecology & Evolution*, 2(10):1661–1672, Oct 2018.

[14] X. Feng and L. Chen. Scsilicon: a tool for synthetic single-cell dna sequencing data generation. *BMC Genomics*, 23(4):359, May 2022.

[15] L. Feuk, A. R. Carson, and S. W. Scherer. Structural variation in the human genome. *Nature Reviews Genetics*, 7(2):85–97, Feb 2006.

[16] J. L. Freeman, G. H. Perry, L. Feuk, R. Redon, S. A. McCarroll, D. M. Altshuler, H. Aburatani, K. W. Jones, C. Tyler-Smith, M. E. Hurles, N. P. Carter, S. W. Scherer, and C. Lee. Copy number variation: New insights in genome diversity. *Genome Research*, 16(8):949–961, 2006.

[17] G. Fudenberg, G. Getz, M. Meyerson, and L. Mirny. High-order chromatin architecture determines the landscape of chromosomal alterations in cancer. *Nature Precedings*, Sep 2011.

[18] R. Gao, S. Bai, Y. C. Henderson, Y. Lin, A. Schalck, Y. Yan, T. Kumar, M. Hu, E. Sei, A. Davis, F. Wang, S. F. Shaitelman, J. R. Wang, K. Chen, S. Moulder, S. Y. Lai, and N. E. Navin. Delineating copy number and clonal substructure in human tumors from single-cell transcriptomes. *Nat Biotechnol*, 39(5):599–608, Jan. 2021.

[19] R. Gao, A. Davis, T. O. McDonald, E. Sei, X. Shi, Y. Wang, P.-C. Tsai, A. Casasent, J. Waters, H. Zhang, F. Meric-Bernstam, F. Michor, and N. E. Navin. Punctuated copy number evolution and clonal stasis in triple-negative breast cancer. *Nature Genetics*, 48(10):1119–1130, Oct 2016.

[20] Y. Gao, X. Ni, H. Guo, Z. Su, Y. Ba, Z. Tong, Z. Guo, X. Yao, X. Chen, J. Yin, Z. Yan, L. Guo, Y. Liu, F. Bai, X. S. Xie, and N. Zhang. Single-cell sequencing deciphers a convergent evolution of copy number alterations from primary to circulating tumor cells. *Genome Research*, 27(8):1312–1322, 2017.

[21] T. Garvin, R. Aboukhalil, J. Kendall, T. Baslan, G. S. Atwal, J. Hicks, M. Wigler, and M. C. Schatz. Interactive analysis and assessment of single-cell copy-number variations. *Nature methods*, 12(11):1058–1060, Nov 2015.

[22] B. Gastman, P. K. Agarwal, A. Berger, G. Boland, S. Broderick, L. H. Butterfield, D. Byrd, P. E. Fecci, R. L. Ferris, Y. Fong, S. L. Goff, M. M. Grabowski, F. Ito, M. Lim, M. T. Lotze, H. Mahdi, M. Malafa, C. D. Morris, P. Murthy, R. I. Neves, A. Odunsi, S. I. Pai, S. Prabhakaran, S. A. Rosenberg, R. Saoud, J. Sethuraman, J. Skitzki, C. L. Slingluff, V. K. Sondak, J. B. Sunwoo, S. Turcotte, C. C. Yeung, and H. L. Kaufman. Defining best practices for tissue procurement in immuno-oncology clinical trials: consensus statement from the society for immunotherapy of cancer surgery committee. *Journal for ImmunoTherapy of Cancer*, 8(2), 2020.

[23] M. Gerstung, N. Eriksson, J. Lin, B. Vogelstein, and N. Beerenwinkel. The temporal order of genetic and pathway alterations in tumorigenesis. *PLOS ONE*, 6(11):1–9, 11 2011.

[24] C. Giguere, H. V. Dubey, V. K. Sarsani, H. Saddiki, S. He, and P. Flaherty. Scsim: Jointly simulating correlated single-cell and bulk next-generation dna sequencing data. *BMC Bioinformatics*, 21(1):215, May 2020.

[25] M. Greaves and C. C. Maley. Clonal evolution in cancer. *Nature*, 481(7381):306–313, Jan 2012.

[26] L. Harbers, F. Agostini, M. Nicos, D. Poddighe, M. Bienko, and N. Crosetto. Somatic copy number alterations in human cancers: An analysis of publicly available data from the cancer genome atlas. *Frontiers in Oncology*, 11, 2021.

[27] L. A. Herzenberg, R. G. Sweet, and L. A. Herzenberg. Fluorescence-activated cell sorting. *Scientific American*, 234(3):108–118, 1976.

[28] R. R. Hudson. Generating samples under a Wright–Fisher neutral model of genetic variation . *Bioinformatics*, 18(2):337–338, 02 2002.

[29] A. Itsara, H. Wu, J. D. Smith, D. A. Nickerson, I. Romieu, S. J. London, and E. E. Eichler. De novo rates and selection of large copy number variation. *Genome Research*, 20(11):1469–1481, 2010.

[30] C. Kim, R. Gao, E. Sei, R. Brandt, J. Hartman, T. Hatschek, N. Crosetto, T. Foukakis, and N. E. Navin. Chemoresistance evolution in triple-negative breast cancer delineated by single-cell sequencing. *Cell*, 173(4):879–893.e13, 2018.

[31] K. A. Knouse, T. Davoli, S. J. Elledge, and A. Amon. Aneuploidy in cancer: Seq-ing answers to old questions. *Annual Review of Cancer Biology*, 1(1):335–354, 2017.

[32] M. Konoshenko, G. Sagaradze, E. Orlova, T. Shtam, K. Proskura, R. Kamyshinsky, N. Yunusova, A. Alexandrova, A. Efimenko, and S. Tamkovich. Total blood exosomes in breast cancer: Potential role in crucial steps of tumorigenesis. *International Journal of Molecular Sciences*, 21(19), 2020.

[33] O. Krijgsman, B. Carvalho, G. A. Meijer, R. D. Steenbergen, and B. Ylstra. Focal chromosomal copy number aberrations in cancer—needles in a genome haystack. *Biochimica et Biophysica Acta (BBA) - Molecular Cell Research*, 1843(11):2698–2704, 2014.

[34] S. Lauer, G. Avecilla, P. Spealman, G. Sethia, N. Brandt, S. F. Levy, and D. Gresham. Single-cell copy number variant detection reveals the dynamics and diversity of adaptation. *PLOS Biology*, 16(12):1–35, 12 2018.

[35] S. Ling, Z. Hu, Z. Yang, F. Yang, Y. Li, P. Lin, K. Chen, L. Dong, L. Cao, Y. Tao, L. Hao, Q. Chen, Q. Gong, D. Wu, W. Li, W. Zhao, X. Tian, C. Hao, E. A. Hungate, D. V. T. Catenacci, R. R. Hudson, W.-H. Li, X. Lu, and C.-I. Wu. Extremely high genetic diversity in a single tumor points to prevalence of non-darwinian cell evolution. *Proceedings of the National Academy of Sciences*, 112(47):E6496–E6505, 2015.

[36] W. Liu, S. Laitinen, S. Khan, M. Vihinen, J. Kowalski, G. Yu, L. Chen, C. M. Ewing, M. A. Eisenberger, M. A. Carducci, W. G. Nelson, S. Yegnasubramanian, J. Luo, Y. Wang, J. Xu, W. B. Isaacs, T. Visakorpi, and G. S. Bova. Copy number analysis indicates monoclonal origin of lethal metastatic prostate cancer. *Nature Medicine*, 15(5):559–565, May 2009.

[37] J. R. MacDonald, R. Ziman, R. K. C. Yuen, L. Feuk, and S. W. Scherer. The Database of Genomic Variants: a curated collection of structural variation in the human genome. *Nucleic Acids Research*, 42(D1):D986–D992, 10 2013.

[38] X. F. Mallory, M. Edrisi, N. Navin, and L. Nakhleh. Assessing the performance of methods for copy number aberration detection from single-cell dna sequencing data. *PLOS Computational Biology*, 16(7):1–24, 07 2020.

[39] X. F. Mallory, M. Edrisi, N. Navin, and L. Nakhleh. Methods for copy number aberration detection from single-cell dna-sequencing data. *Genome Biol.*, 21(1):208, Aug 2020.

[40] X. F. Mallory and L. Nakhleh. SimSCSnTree: a simulator of single-cell DNA sequencing data. *Bioinformatics*, 38(10):2912–2914, 03 2022.

[41] R. Meza, J. Jeon, S. H. Moolgavkar, and E. G. Luebeck. Age-specific incidence of cancer: Phases, transitions, and biological implications. *Proceedings of the National Academy of Sciences*, 105(42):16284–16289, 2008.

[42] J. S. Michaelson, M. Silverstein, J. Wyatt, G. Weber, R. Moore, E. Halpern, D. B. Kopans, and K. Hughes. Predicting the survival of patients with breast carcinoma using tumor size. *Cancer*, 95(4):713–723, 2002.

[43] D. C. Minussi, M. D. Nicholson, H. Ye, A. Davis, K. Wang, T. Baker, M. Tarabichi, E. Sei, H. Du, M. Rabbani, C. Peng, M. Hu, S. Bai, Y.-w. Lin, A. Schalck, A. Multani, J. Ma, T. O. McDonald, A. Casasent, A. Barrera, H. Chen, B. Lim, B. Arun, F. Meric-Bernstam, P. Van Loo, F. Michor, and N. E. Navin. Breast tumours maintain a reservoir of subclonal diversity during expansion. *Nature*, 592(7853):302–308, Apr 2021.

[44] N. Navin, J. Kendall, J. Troge, P. Andrews, L. Rodgers, J. McIndoo, K. Cook, A. Stepansky, D. Levy, D. Esposito, L. Muthuswamy, A. Krasnitz, W. R. McCombie, J. Hicks, and M. Wigler. Tumour evolution inferred by single-cell sequencing. *Nature*, 472(7341):90–94, Apr 2011.

[45] A. Niida, T. Hasegawa, H. Innan, T. Shibata, K. Mimori, and S. Miyano. A unified simulation model for understanding the diversity of cancer evolution. *PeerJ*, 8:e8842, Apr. 2020.

[46] A. Niida, W. M. Iwasaki, and H. Innan. Neutral Theory in Cancer Cell Population Genetics. *Molecular Biology and Evolution*, 35(6):1316–1321, 04 2018.

[47] B. Nowakowska. Clinical interpretation of copy number variants in the human genome. *Journal of applied genetics*, 58(4):449–457, Nov 2017.

[48] R. L. Pollex and R. A. Hegele. Copy number variation in the human genome and its implications for cardiovascular disease. *Circulation*, 115(24):3130–3138, 2007.

[49] D. Posada. CellCoal: Coalescent Simulation of Single-Cell Sequencing Samples. *Mol. Biol. Evol.*, 37(5):1535–1542, 02 2020.

[50] T. Saito, A. Niida, R. Uchi, H. Hirata, H. Komatsu, S. Sakimura, S. Hayashi, S. Nambara, Y. Kuroda, S. Ito, H. Eguchi, T. Masuda, K. Sugimachi, T. Tobo, H. Nishida, T. Daa, K. Chiba, Y. Shiraishi, T. Yoshizato, M. Kodama, T. Okimoto, K. Mizukami, R. Ogawa, K. Okamoto, M. Shuto, K. Fukuda, Y. Matsui, T. Shimamura, T. Hasegawa, Y. Doki, S. Nagayama, K. Yamada, M. Kato, T. Shibata, M. Mori, H. Aburatani, K. Murakami, Y. Suzuki, S. Ogawa, S. Miyano, and K. Mimori. A temporal shift of the evolutionary principle shaping intratumor heterogeneity in colorectal cancer. *Nature Communications*, 9(1):2884, Jul 2018.

[51] M. Secrier, X. Li, N. de Silva, M. D. Eldridge, G. Contino, J. Bornschein, S. MacRae, N. Grehan, M. O'Donovan, A. Miremadi, T.-P. Yang, L. Bower, H. Chettouh, J. Crawte, N. Galeano-Dalmau, A. Grabowska, J. Saunders, T. Underwood, N. Waddell, A. P. Barbour, B. Nutzinger, A. Achilleos, P. A. W. Edwards, A. G. Lynch, S. Tavaré, R. C. Fitzgerald, A. Noorani, R. F. Elliott, J. Weaver, C. Ross-Innes, L. Smith, Z. Abdullahi, R. de la Rue, A. Cluroe, S. Malhotra, R. Hardwick, H. Ford, M. L. Smith, J. Davies, R. Turkington, S. J. Hayes, Y. Ang, S. R. Preston, S. Oakes, I. Bagwan, V. Save, R. J. E. Skipworth, T. R. Hupp, J. R. O'Neill, O. Tucker, P. Taniere, F. Noble, J. Owsley, L. Lovat, R. Haidry, V. Eneh, C. Crichton, H. Barr, N. Shepherd, O. Old, J. Lagergren, J. Gossage, A. Davies, F. Chang, J. Zylstra, G. Sanders, R. Berrisford, C. Harden, D. Bunting, M. Lewis, E. Cheong, B. Kumar, S. L. Parsons, I. Soomro, P. Kaye, P. Collier, L. Igali, I. Welch, M. Scott, S. Sothi, S. Suortamo, S. Lishman, D. Beardsmore, H. E. Francies, M. J. Garnett, J. V. Pearson, K. Nones, A.-M. Patch, S. M. Grimmond, the Oesophageal Cancer Clinical, and M. S. O. Consortium. Mutational signatures in esophageal adenocarcinoma define etiologically distinct subgroups with therapeutic relevance. *Nature Genetics*, 48(10):1131–1141, Oct 2016.

[52] S. P. Shah, X. Xuan, R. J. DeLeeuw, M. Khojasteh, W. L. Lam, R. Ng, and K. P. Murphy. Integrating copy number polymorphisms into array CGH analysis using a robust HMM. *Bioinformatics*, 22(14):e431–e439, 07 2006.

[53] A. Shlien and D. Malkin. Copy number variations and cancer. *Genome medicine*, 1(6):62–62, Jun 2009.

[54] A. Sottoriva, H. Kang, Z. Ma, T. A. Graham, M. P. Salomon, J. Zhao, P. Marjoram, K. Siegmund, M. F. Press, D. Shibata, and C. Curtis. A big bang model of human colorectal tumor growth. *Nature Genetics*, 47(3):209–216, Mar 2015.

[55] A. Srivatsa, H. Lei, and R. Schwartz. A clonal evolution simulator for planning somatic evolution studies. In M. S. Bansal, Z. Cai, and S. Mangul, editors, *Bioinformatics Research and Applications*, pages 229–242, Cham, 2022. Springer Nature Switzerland.

[56] R. Sun, Z. Hu, and C. Curtis. Big bang tumor growth and clonal evolution. *Cold Spring Harb Perspect Med*, 8(5), May 2018.

[57] A. M. Taylor, J. Shih, G. Ha, G. F. Gao, X. Zhang, A. C. Berger, S. E. Schumacher, C. Wang, H. Hu, J. Liu, A. J. Lazar, Cancer Genome Atlas Research Network, A. D. Cherniack, R. Beroukhim, and M. Meyerson. Genomic and functional approaches to understanding cancer aneuploidy. *Cancer Cell*, 33(4):676–689.e3, Apr. 2018.

[58] R. Uchi, Y. Takahashi, A. Niida, T. Shimamura, H. Hirata, K. Sugimachi, G. Sawada, T. Iwaya, J. Kurashige, Y. Shinden, T. Iguchi, H. Eguchi, K. Chiba, Y. Shiraishi, G. Nagae, K. Yoshida, Y. Nagata, H. Haeno, H. Yamamoto, H. Ishii, Y. Doki, H. Iinuma, S. Sasaki, S. Nagayama, K. Yamada, S. Yachida, M. Kato, T. Shibata, E. Oki, H. Saeki, K. Shirabe, Y. Oda, Y. Maehara, S. Komune, M. Mori, Y. Suzuki, K. Yamamoto, H. Aburatani, S. Ogawa, S. Miyano, and K. Mimori. Integrated multiregional analysis proposing a new model of colorectal cancer evolution. *PLOS Genetics*, 12(2):1–24, 02 2016.

[59] M. Upadhyay, V. H. da Silva, H.-J. Megens, M. H. P. W. Visker, P. Ajmone-Marsan, V. A. Bâlteanu, S. Dunner, J. F. Garcia, C. Ginja, J. Kantanen, M. A. M. Groenen, and R. P. M. A. Crooijmans. Distribution and functionality of copy number variation across european cattle populations. *Frontiers in Genetics*, 8, 2017.

[60] E. I. Velazquez-Villarreal, S. Maheshwari, J. Sorenson, I. T. Fiddes, V. Kumar, Y. Yin, M. G. Webb, C. Catalanotti, M. Grigorova, P. A. Edwards, J. D. Carpten, and D. W. Craig. Single-cell sequencing of genomic dna resolves sub-clonal heterogeneity in a melanoma cell line. *Communications Biology*, 3(1):318, Jun 2020.

[61] B. Waclaw, I. Bozic, M. E. Pittman, R. H. Hruban, B. Vogelstein, and M. A. Nowak. A spatial model predicts that dispersal and cell turnover limit intratumour heterogeneity. *Nature*, 525(7568):261–264, Sep 2015.

[62] R. Wang, D.-Y. Lin, and Y. Jiang. Scope: A normalization and copy-number estimation method for single-cell dna sequencing. *Cell Systems*, 10(5):445–452.e6, 2020.

[63] M. J. Williams, B. Werner, C. P. Barnes, T. A. Graham, and A. Sottoriva. Identification of neutral tumor evolution across cancer types. *Nature Genetics*, 48(3):238–244, Mar 2016.

[64] H. Yang, B. Lu, L. H. Lai, A. H. Lim, J. J. S. Alvarez, and W. Zhai. PSiTE: a Phylogeny guided Simulator for Tumor Evolution. *Bioinformatics*, 35(17):3148–3150, 01 2019.

[65] Z. Yu, F. Du, X. Sun, and A. Li. SCSsim: an integrated tool for simulating single-cell genome sequencing data. *Bioinformatics*, 36(4):1281–1282, 09 2019.

[66] S. Zaccaria and B. J. Raphael. Characterizing allele- and haplotype-specific copy numbers in single cells with chisel. *Nature Biotechnology*, 39(2):207–214, Feb 2021.

[67] T. I. Zack, S. E. Schumacher, S. L. Carter, A. D. Cherniack, G. Saksena, B. Tabak, M. S. Lawrence, C.-Z. Zhang, J. Wala, C. H. Mermel, C. Sougnez, S. B. Gabriel, B. Hernandez, H. Shen, P. W. Laird, G. Getz, M. Meyerson, and R. Beroukhim. Pan-cancer patterns of somatic copy number alteration. *Nature Genetics*, 45(10):1134–1140, Oct 2013.
